# Interventions to improve the nutritional status of children under 5 years in Ethiopia: a systematic review

**DOI:** 10.1017/S1368980023002410

**Published:** 2023-12

**Authors:** Kedir Y Ahmed, Felix Akpojene Ogbo, Teketo Kassaw Tegegne, Hazel Dalton, Amit Arora, Allen G Ross

**Affiliations:** 1 Rural Health Research Institute, Charles Sturt University, Orange, NSW, Australia; 2 Translational Health Research Institute, Western Sydney University, Campbelltown, NSW, Australia; 3 Riverland Academy of Clinical Excellence (RACE), Riverland Mallee Coorong Local Health Network, SA Health, Government of South Australia, Berri, SA, Australia; 4 Institute for Physical Activity and Nutrition, Deakin University, Geelong, VIC, Australia; 5 School of Medicine and Public Health, University of Newcastle, Orange, NSW, Australia; 6 School of Health Sciences, Western Sydney University, Campbelltown Campus, NSW, Australia; 7 Oral Health Services, Sydney Local Health District and Sydney Dental Hospital, NSW Health, Surry Hills, NSW, Australia; 8 Discipline of Child and Adolescent Health, Sydney Medical School, Faculty of Medicine and Health, The University of Sydney, Westmead, NSW, Australia; 9 Health Equity Laboratory, Campbelltown, NSW, Australia

**Keywords:** Nutritional interventions, Malnutrition, Stunting, Children, Pregnant women, Ethiopia

## Abstract

**Objective:**

To conduct a systematic review of experimental or quasi-experimental studies that aimed to improve the nutritional status of children under 5 years of age in Ethiopia.

**Design:**

Embase, MEDLINE/PubMed, Cumulative Index to Nursing and Allied Health Literature (CINAHL), PsychINFO, and Academic Search Database were used to locate peer-reviewed studies, and Google Scholar and Open Dissertation were used to locate grey literatures. All searches were conducted between 2000 and November 2022.

**Setting:**

Ethiopia.

**Participants:**

Pregnant women and mothers with children aged 0–59 months.

**Results:**

Ten cluster randomised controlled trials (RCT), six quasi-experimental studies and two individual RCT were included. Out of the identified eighteen studies, three studies targeted pregnant mothers. Our findings showed that almost two-thirds of published interventions had no impact on childhood stunting and wasting, and more than half had no impact on underweight. Some behaviour change communication (BCC) interventions, food vouchers, micronutrient supplementation and quality protein maize improved stunting. Similarly, BCC and fish oil supplementation showed promise in reducing wasting, while BCC and the provision of quality protein maize reduced underweight. Additionally, water, sanitation and hygiene (WaSH) interventions provided to pregnant mothers and children under 2 years of age were shown to significantly reduce childhood stunting.

**Conclusion:**

Future childhood nutritional interventions in Ethiopia should consider adopting an integrated approach that combines the positive effects of interdependent systems such as BCC, food supplemental programmes (e.g. boosting protein and micronutrients), health interventions (e.g. strengthening maternal and childcare), WaSH and financial initiatives (e.g. monetary support and income schemes).

Ending all forms of childhood malnutrition, including stunting, wasting and underweight, is a global priority^(1,2)^. In 2021, a multi-agency report estimated that the global prevalence of stunting had decreased from approximately 33·0 % in 2000 to 22·0 % in 2020, and wasting had improved slightly from about 8·0 % to 7·0 % between 2012 and 2020^(3)^. Despite these improvements, there exist global childhood nutrition disparities within and between countries^(4–6)^. Some reports suggest that these improvements are not enough to successfully achieve the global nutrition targets of ending malnutrition by 2030^(1,2,7)^. In sub-Saharan African countries, including Ethiopia, where there is a high burden of stunted, wasted and underweight children, the possibility of not attaining the nutrition targets is even greater^(3,8)^.

Ethiopia is the second most populous country in Africa^(9,10)^, but also a landlocked country since the 1993 secession of Eritrea^(9)^. In the past two decades, Ethiopia has seen remarkable progress in reducing the burden of under-five mortality (from 166 deaths per 1000 in 2000 to 67 deaths per 1000 in 2016) and stunting (from 58·0 % in 2000 to 37·0 % in 2019)^(11–14)^. Although these improvements are important and commendable, it is concerning that one in fifteen children die before the age of 5 years^(12)^, and undernutrition remains a major contributor (28 %) to these deaths in the country^(15)^. Furthermore, there is an ongoing strain on the Ethiopian economy, from natural and man-made disasters (e.g. conflicts and internal displacements)^(16,17)^, which subsequently increases the vulnerability to low-yield crop production, food insecurity^(18,19)^ and malnutrition^(20–22)^.

Building on the Sustainable Development Goals^(2)^ and the UN Decade of Action on Nutrition (2016–2025)^(1)^, Ethiopia has implemented several programmes to eliminate childhood malnutrition. In 2015, the ‘Seqota’ Declaration was launched ‘to end stunting in children under 2 years by 2030 in Ethiopia’^(23)^. The Seqota Declaration employed a multisectoral approach and comprises three phases – the Innovation, Expansion, and Scale-up phases, and three pathways – nutrition-specific, nutrition-sensitive and infrastructure interventions^(23)^. To provide a national framework for the coordinated implementation of nutritional interventions, the National Nutrition Program (NNP-II) was developed in 2016. This programme aimed to reduce undernutrition and micronutrient deficiencies, improve maternal and child health, and enhance the capacity of the health system to deliver quality nutrition services^(24)^.

Furthermore, in 2018, the National Food and Nutrition Policy was endorsed, which addresses the immediate and underlying causes of malnutrition in Ethiopia, focusing on the prevention and treatment of undernutrition and micronutrient deficiencies^(25)^. In 2021, the Ethiopian Health Sector Transformation Plan (HSTP-II) was launched to improve the overall population’s health outcomes, including children’s nutritional outcomes, by strengthening the maternal health system and increasing access to quality health services^(26)^. These programmes and policies aim to enhance the effectiveness and sustainability of Ethiopia’s efforts to combat childhood malnutrition.

Despite the implementation of these strategic policy interventions to improve the nutritional and growth outcomes of Ethiopian children, no systematic review has examined the impacts of these interventions. Information on the effectiveness of these interventions is essential in understanding current gains, gaps and future priorities, where decision-makers and public health practitioners can increase efforts to improve the nutritional status of children. Accordingly, the main aim of this systematic review is to investigate the impacts of community-based and health facility interventions that set out to address the nutritional status of under 5 years of age children in Ethiopia.

## Methods

This systematic review was reported following the 2020 Preferred Reporting Items for Systematic Reviews and Meta-Analyses (PRISMA) statement^(27)^ (see online Supplemental file 1)

### Eligibility criteria

The eligible studies for this study needed to fulfil the following criteria:

1. **Study design**: Experimental or quasi-experimental studies, including randomised controlled trials (RCTs), non-RCTs, before and after studies, and interrupted time-series studies, with or without comparison groups or clusters;
2. **Interventions**: Community-based and health facility interventions targeted pregnant women and mothers with children under 5 years of age. These interventions included nutritional education and counselling, interpersonal communication, mass media campaigns, nutrition-sensitive agricultural activities, group recipe demonstration sessions, micronutrient supplementation, and the strengthening of health facilities. Our study excluded interventional studies on vulnerable groups, such as pregnant women and children, in emergencies;
3. **Outcome measures**: Measurement of child anthropometric outcomes, such as stunting (height/length-for-age *z*-scores), wasting (weight-for-length/height *z*-scores) and underweight (weight-for-age *z*-score), in continuous or dichotomous forms; and
4. **Language and location**: The studies needed to be published in the English language and conducted in Ethiopia from the year 2000 to November 2022.

The primary reason for selecting this period was to analyse the impacts of global initiatives, such as the Millennium Development Goals (MDG)^(28)^ and the current Sustainable Development Goals, on the nutritional status of children in Ethiopia^(29)^.

### Information sources and search strategy

A three-stage search strategy was implemented to locate both peer-reviewed articles and grey literatures, consistent with prior systematic reviews^(30–32)^. In stage one, we conducted a manual search to check for previously published systematic reviews of interventions on childhood malnutrition in Ethiopia. In stage two, an initial search was conducted using PubMed ID to generate standard and key terms through the online tool ‘Yale MeSH Analyser’ (https://mesh.med.yale.edu/). In the last stage, a full search was performed on five computerised electronic databases (including Embase/Ovid, PsychINFO/EBSCOhost, Academic Search database/EBSCOhost, MEDLINE(Ovid)/PubMed and Cumulative Index to Nursing and Allied Health Literature (CINAHL)/ EBSCOhost) to locate peer-reviewed articles. Whereas Google Scholar and Open Dissertation were used to locate grey literatures. The search strategy was devised with the Population Intervention Comparator Outcome (PICO) criteria, and all identified index and keywords terms were slightly adapted for each of the information sources. The initial and the top-up searches were conducted in August 2022 and November 2022, respectively. Supplemental file 2 provides the search strategy for the MEDLINE (Ovid) database.

The search terms/keywords used included:

**Term 1 (Population)**: child, infant, newborn, baby, neonate, perinatal, postnatal, kid, toddler, young child, paediatric, mother, female, women and caregiver.

### Term 2 (Interventions)

- **Education and counselling**: breast-feeding promotion, breast-feeding support, breast-feeding education, health education, health promotion, nutrition education, food education, parent education, mother education, counselling, and nutritional counselling.
- **Social behavioural change communication**: health behaviour, health-related behaviour, behaviour change, communication, interpersonal communication, information education communication, behaviour change communication (BCC), social change, social movement, social mobilisation, social behaviour, social network, peer group, advocacy, advocacy group, mass communication, mass media, print media, mobile phone, mHealth, eHealth, internet, radio, social media, television, text message and social market.
- **Community-based approaches**: baby-friendly community initiative, community programme, community project, home visit, community health action, community health service, community healthcare, community intervention, community engagement, community leader, community mobilisation, demonstration, cooking demonstration, community role play, model breast-feeding community, community health worker and health extension worker.
- **Facility-based approaches**: maternal care, child healthcare, child health service, paediatric healthcare, neonatal care, newborn care, rooming-in-care, newborn nursery, essential nutrition action, caregiver contact, baby-friendly hospital initiative, BFHI, antenatal care, postnatal care, Kangaroo care and skin-to-skin contact.
- **Intervention designs**: effectiveness, impact, evaluation study, programme evaluation, healthcare programme, project, health project, experimental study, interventional study, quasi-experimental study, RCT, clinical trial, cluster-randomised trial, time-series study, control, placebo, comparison and usual care.

**Term 3 (Context)**: Ethiopia.

**Term 4 (Outcomes)**: stunting, wasting, undernutrition, malnutrition, thinness, hunger, growth disorders, developmental disorders, nutritional disorders and nutritional status.

### Study selection

All documents retrieved from the search database were exported to EndNote library version 20.2.1 (The EndNote Team, Philadelphia) for initial title/abstract screening^(33)^. Two reviewers (K.Y.A. and F.A.O.) independently conducted the title and abstract screening using the pre-formed inclusion and exclusion criteria. Articles passing the initial screening were subjected to a full-text review. The full text of the included studies was then checked independently by two reviewers (K.Y.A. and F.A.O.) using the eligibility criteria. We excluded studies that did not meet the eligibility criteria and the reasons for the exclusion of studies recorded and reported. Disagreements between the two independent reviewers were resolved by consensus and arbitration with the third reviewer (T.K.T.).

### Data collection process and data items

Using an adapted form from the Cochrane Pregnancy and Childbirth Group for Systematic Reviews^(34)^, K.Y.A. carried out the data extraction and F.A.O. independently verified the extracted data. Eligible studies were identified using the following information: first author, publication year, study design and setting, study participants, sample size, geographical region, intervention components, location of intervention delivery, target group, intervention period, outcome measures, statistical analysis, and results. For eligible studies with incomplete information, a total of two contact attempts were made, and if no response was received, only the information available was used.

### Risk of bias assessment

Two reviewers (K.Y.A. and T.K.T.) independently checked eligible studies for selection, performance, attrition, detection and reporting biases. The Cochrane risk of bias tools for randomised trials (ROB 2.0) for individual RCT, the revised ROB 2.0 CRT for cluster RCT^(35,36)^ and the risk of bias in non-randomised studies – of interventions (ROBINS-I) for quasi-experimental studies^(37)^ were used for the risk of bias assessment. Cluster-randomised trials were assessed for six domains (i.e. bias from randomisation process, identification or recruitment bias, deviations from intended interventions, missing data, measurement of outcomes and selection of reported result), and quasi-experimental studies were assessed for seven domains (i.e. confounding, selection of participants, classification of interventions, deviations from intended interventions, missing data, measurement of outcomes and selection of reported result). For RCT, the overall risk of bias included low risk, some concerns and high risk^(35,36)^. Likewise, for quasi-experimental studies, the overall risk of bias included low, moderate, serious and critical risk of bias^(37)^.

### Results synthesis

Given the heterogeneity of participants, intervention duration, type of interventions and outcome measures, we narratively reported the author’s reports of effect size measures for each study. Effect size measures included a comparison between experimental and control groups (e.g. OR), the difference in proportion between pre- and post-outcome measures (e.g. change in proportion) and the difference-in-difference (DID) measures. As appropriate, 95 % CI and *P*-values were also obtained from eligible studies.

## Results

### Description of studies

**Fig. 1:**
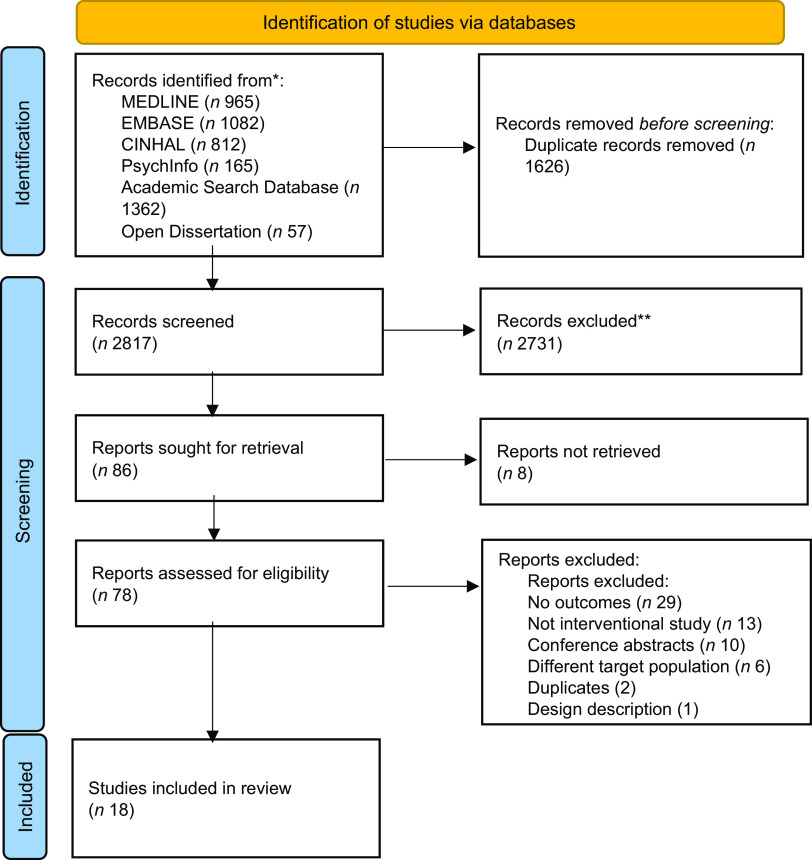
The 2020 Preferred Reporting Items for Systematic Reviews and Meta-Analyses (PRISMA) flow chart for the selection of eligible studies^(74)^

Our database search identified 4443 studies, and 1626 duplicates were removed from this subset of studies. Out of 2817 articles screened for titles and abstracts, seventy-eight were retained for full-text eligibility checks and eighteen articles were found to be eligible for this study (Fig. 1). Supplementary file 3 presents the list of excluded studies.

The included studies were ten cluster RCT^(38–47)^, six quasi-experimental studies^(48–53)^ and two RCT^(46,54)^. The most common location for the delivery of interventions was Oromia Region^(38,40,42,45–47,49,50,54)^, followed by Amhara Region^(39,42–44,48,53)^. Additionally, there were studies conducted in the Southern Nations Nationalities and Peoples (SNNP)^(41,42,46,50–52)^ and Tigray regions^(52)^. In the review, no interventional studies were published for the regions of Afar, Somali, Harari, Gambella, and Benishangul, or Addis Ababa and Dire Dawa city administrations.

The most common type of interventions were BCC such as nutritional education^(40,48,49,51,53)^, educational materials^(38,39,44,49)^, cooking demonstrations^(38,39,41,45,49,51)^, group discussion forums^(39,40,49)^, home visits and supervision^(39,41,45,48)^, breast-feeding support^(40)^ and community mobilisation^(44,52)^. Additionally, there were interventions involving food vouchers^(47)^, micronutrient supplementations^(43,50,54)^, provision of chicken^(42)^ and quality protein maize (maize varieties fortified with elevated lysine and tryptophan levels)^(46)^. A publication by Akalu *et al.* reported two different studies in a single article^(46)^. Table 1 shows the summary of eligible studies.

**Table 1:** Summary of eligible studies

Authors	Design	Participants	Region	Intervention components	Location of intervention delivery	Target group	Intervention period	Measured outcomes
Mekonnen *et al.*, 2022^(48)^	Quasi-experimental	Intervention group Baseline = 170 Endline = 270	Amhara	Nutrition education packages: • education and counselling of mothers/caregivers • training of community-based nutrition mentors (HEW, WDA, ADA and farmers) • home visits and supervision • provision of vegetable seeds and egg-laying pullets	Community-based intervention	Mothers/caregivers with < 12 months child	12 months	Stunting Wasting Underweight
Habtmiriam *et al*., 2022^(38)^	Cluster-randomised trial	Intervention group Baseline = 506 Endline = 406 Control group Baseline = 506 Endline = 309	Oromia	Behavioural change communication interventions: • leaflets, pictures and posters • assisting child feeding activities • demonstration	Community-based interventions	Mothers/care givers with 6–59 months children	15 months	Stunting Wasting Underweight
Bidira *et al*., 2022^(49)^	Quasi-experimental	Intervention group Baseline = 283 Endline = 272 Control group Baseline = 286 Endline = 277	Oromia	Nutrition education packages: • healthy diet awareness • nutrition and hygiene • group discussions, lectures and roleplays • demonstrations	Community-based interventions	Mothers/care givers with 24–59 months children	9 months	Stunting Wasting Underweight
Ayalew *et al*., 2020^(39)^	Cluster-randomised trial	Intervention group Baseline = 306 Endline = 272 Control group Baseline = 306 Endline = 282	Amhara	Behavioural change communication interventions: • complementary feeding messages • cooking demonstrations • posters • group training of mothers/caregivers • home visits	Community-based interventions	Mothers/care givers with infants less than 6 months	9 months	Stunting Wasting Underweight
Han *et al.*, 2021^(47)^	Cluster-randomised trial	BCC group Baseline = 101 Endline = 101 Voucher group Baseline = 96 Endline = 96 BCC + voucher group Baseline = 154 Endline = 154 Control group Baseline = 290 Endline = 290	Oromia	Behavioural change communication interventions: • nutritional education sessions • videos and visual aids • role plays and cooking sessions Food voucher interventions: • 200 ETB (10 USD) per month for food items	Community-based interventions	Mothers/care givers with children 6 to 20 months	16 weeks	Stunting Wasting
Abdulahi *et al*., 2020^(40)^	Cluster-randomised trial	Intervention group Baseline = 249 Endline = 212 Control group Baseline = 219 Endline = 197	Oromia	Breast-feeding education and peer support: • breast-feeding education • demonstrations on proper breast-feeding positions and attachments • nutritional education during pregnancy • peer support	Community-based interventions	Pregnant women	From pregnancy to 13 months postpartum	Stunting Wasting Underweight
Teshome *et al*., 2020^(41)^	Cluster-randomised trial	Intervention group Baseline = 386 Endline = 307 Control group Baseline = 386 Endline = 314	Sidama	Behavioural change communication interventions using health belief model and theory of planned behaviour: • recipe demonstrations • counselling using home visits	Community-based interventions	Mothers/care givers with 6–15 months children	9 months	Stunting Wasting Underweight
Passarelli *et al*., 2020^(42)^	Cluster-randomised trial	ACGG group Baseline = 391 Endline = 311 ACGG + ATONU group Baseline = 287 Endline = 263 Control group Baseline = 272 Endline = 255	Amhara Oromia SNNP Tigray	ACGG interventions: • 25 vaccinated chickens provided for households ACGG + ATONU group: • 25 vaccinated chickens provided for households • promotion of home gardening • provision of fruit and vegetable seeds • behavioural change communication	Community-based interventions	Mothers/care givers with 0–35 months children	14 months	Stunting Wasting Underweight
Mohammed *et al*., 2020^(43)^	Cluster-randomised trial	Intervention group Baseline = 651 Endline = 536 Control group Baseline = 569 Endline = 488	Amhara	Community-level salt monitoring and control: • ensure distributers supply as iodised salt • banning of non-iodised salt • social marketing of iodised salt usage	Community-based interventions	Pregnant women	From pregnancy to 13 months postpartum	Stunting Wasting Underweight
Kang *et al.*, 2017^(45)^	Cluster-randomised trial	Intervention group Baseline = 914 Endline = 711 Control group Baseline = 876 Endline = 684	Oromia	Complementary feeding programme: • group nutrition sessions • cooking demonstrations • home visits • supervision and training of community workers	Community-based interventions	Mothers/care givers with 6–12 months children	12 months	Stunting Wasting Underweight
Kim *et al*., 2019^(44)^	Cluster-randomised evaluation	Intervention group Baseline = 1328 Endline = 1360 Control group Baseline = 1318 Endline = 1360	Amhara	Behavioural change communication interventions: • interpersonal communication • community mobilisation • mass media campaign	Community-based interventions	Mothers/care givers with 6–24 months children	24 months	Stunting Wasting Underweight
Samuel *et al*., 2018^(50)^	Quasi-experimental	Intervention group Baseline = 1172 Endline = 1025 Control group Baseline = 1137 Endline = 1052	Oromia SNNP	Intervention (multiple micronutrient powder [MNP]): • low-dose iron MNP (30 sachets/two months)	Community-based interventions	Mothers/care givers with 6–12 months children	12 months	Stunting Wasting Underweight
Argaw *et al*., 2018^(54)^	RCT	Intervention group Baseline = 90 Endline = 83 Placebo group Baseline = 91 Endline = 86	Oromia	Intervention group: • both mother and child received fish oil supplementation	Community-based interventions	Mothers/care givers with 6–12 months children	12 months intervention	Stunting Wasting
Mulualem *et al*., 2016^(51)^	Quasi-experimental	Intervention group Baseline = 80 Endline = 80 Control group Baseline = 80 Endline = 80	SNNP	Behavioural change communication interventions: • nutrition education sessions • health belief model • recipe demonstrations	Community-based interventions	Mothers/care givers with 6–18 months children	6 months intervention	Stunting Wasting Underweight
Kim *et al*., 2016^(52)^	Repeated cross-sectional	Intervention group Baseline = 1481 Endline = 1485	SNNP Tigray	Social and behavioural change communication interventions: • interpersonal communication • community mobilisation • mass media campaign	Community-based interventions	Mothers/care givers with 0–59 months children	4 years	Stunting Wasting Underweight
Akalu *et al*., 2010^(46)^	Cluster-randomised trial	Intervention (quality protein maize) Baseline = 73 Endline = 73 Control (conventional maize) Baseline = 78 Endline = 78	SNNP Oromia	Intervention group: • received seed of quality protein maize (BHQP 542) Control group: • conventional maize quality (BH 140)	Community-based interventions	Households with 5–29 months children	13 months	Stunting Wasting Underweight
Akalu *et al.*, 2010^(46)^	RCT	Intervention (quality protein maize) Baseline = 106 Endline = 106 Control (conventional maize) Baseline = 105 Endline = 105	SNNP Oromia	Intervention group: • received seed of quality protein maize (BHQP 542) Control group: • conventional maize quality (BH 140)	Community-based interventions	Mother/caregivers with children aged 7 to 56 months	11 months	Stunting Wasting Underweight
Fenn *et al*., 2011^(53)^	Repeated cross-sectional	Intervention Baseline = 1428 Endline = 1036 Control (conventional maize) Baseline = 1081 Endline = 683	Amhara	Intervention group: • nutrition and health education for pregnant mothers and children • water, sanitation and hygiene interventions • education on diarrhoea causes and treatment • education on immunisation	Community-based interventions	Pregnant mothers and children less than 2 years	5 years impact evaluation	Stunting

BCC, behaviour change communication; ACGG, African Chicken Genetic Grains; ATONU, agriculture to nutrition; SNNP, Southern Nations Nationalities and Peoples; RCT, randomised controlled trials.

### Risk of bias in included studies

**Fig. 2:**
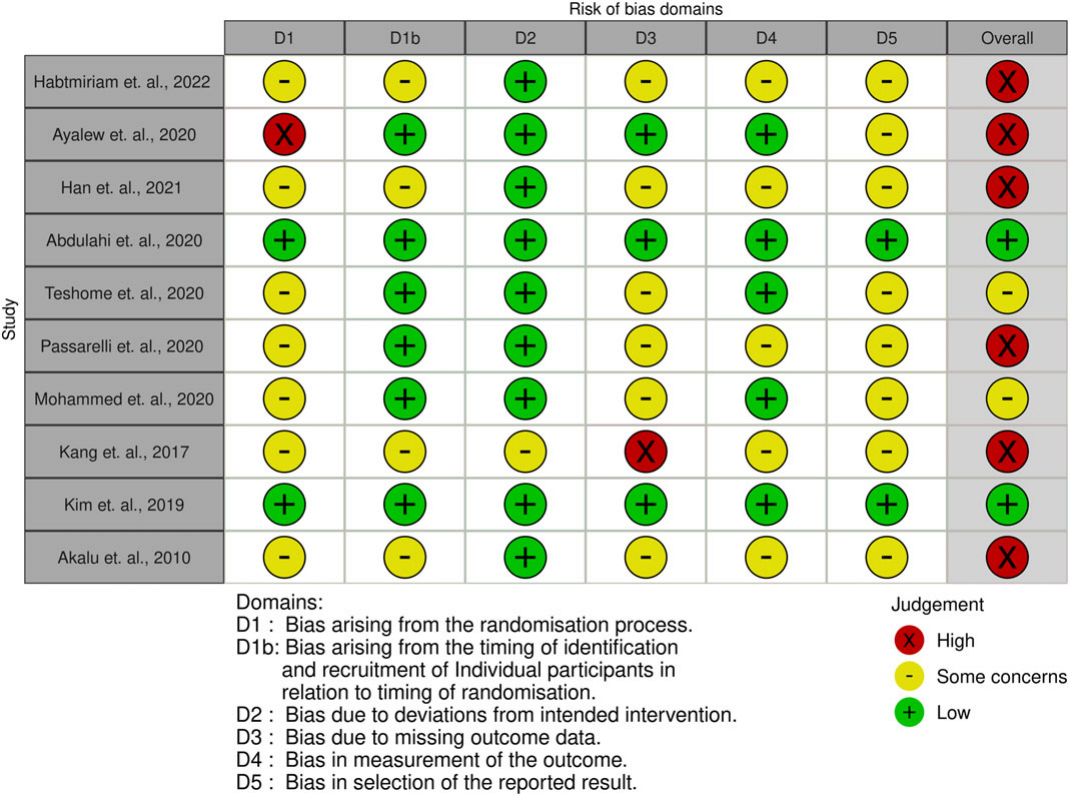
Risk of bias traffic light plot for cluster RCT: review authors’ judgements about each risk of bias item for each study

**Fig. 3:**
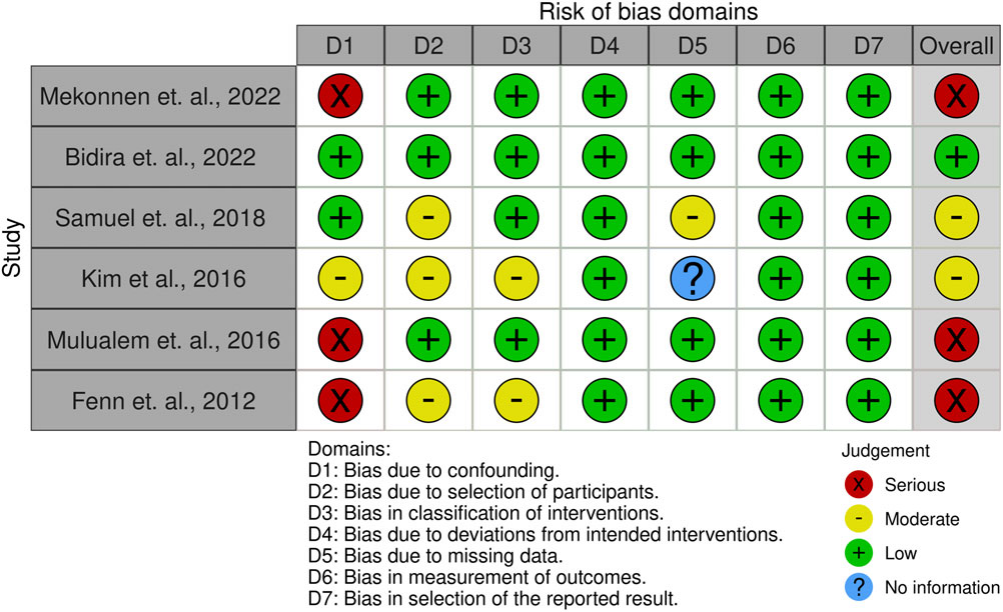
Risk of bias traffic light plot for quasi-experimental studies: review authors’ judgements about each risk of bias item for each study

**Fig. 4:**
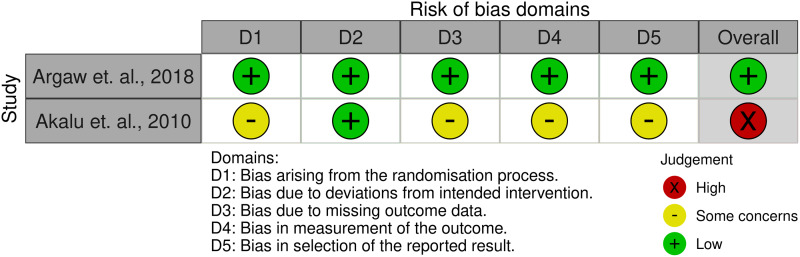
Risk of bias traffic light plot for individual RCT: review authors’ judgements about each risk of bias item for each study

Of the ten cluster RCT, six had a high risk of bias^(38,39,42,45–47)^ and two studies had a low risk of bias^(40,44)^ (Fig. 2). Similarly, of the six quasi-experimental studies, three studies had a serious risk of bias^(48,51,53)^, while two had a moderate risk of bias^(50,52)^ (Fig. 3). One of the two RCT was judged to have a high risk of bias^(46)^ (Fig. 4).

### Impact of interventions on stunting/height-for-age Z-score

This review included eighteen eligible studies, three of which focused on pregnant women, while the remaining fifteen studies targeted postpartum mothers and children aged 0–59 months. The interventions for pregnant women included breast-feeding education and peer support^(40)^, community-level salt monitoring and control^(43)^, and water, sanitation and hygiene (WaSH) programmes^(53)^. However, except for WaSH interventions that targeted pregnant mothers and children under 2 years of age in the form of protected water supply and sanitation education^(53)^, none of the studies targeting pregnant women demonstrated a significant impact on stunting.

Of the fifteen interventional studies implemented on postpartum mothers and children aged 0–59 months, only five studies showed significant impacts of the interventions. Mekonnen *et al.* reported improvements in children’s length-for-age *z*-score through nutritional education interventions for mothers/caregivers of infants aged less than 1 year (MD = −0·73; 95 % CI (−1·40, −0·06), *P*-value = 0·034)^(48)^. A 16-week combination of BCC and food vouchers for mothers/caregivers of children aged 6–20 months resulted in a significant decrease in childhood stunting (percentage change in proportion = 9·7 %, *P*-value < 0·05). However, separate interventions of BCC and food vouchers did not have impacts on stunting^(47)^. Kang *et al.* found improvements in the height-for-age z-score of children with a 12-month complementary feeding programme (including group nutritional sessions and cooking demonstrations for mothers, and supervision and training of health workers) for mothers/caregivers with children aged 6–12 months (MD = 0·32; 95 % CI (0·08, 0·56), *P*-value < 0·001)^(45)^ (Table 2).

**Table 2:** Summary of evidence from review studies

Studies	Statistical analysis	Stunting/HAZ	Wasting/WHZ	Underweight/WAZ
Mekonnen *et al*., 2022^(48)^	Mean change in *Z*-scores	Baseline *v*. Endline (MD = −0·73; 95 % CI (−1·40, −0·06), *P*-value = 0·034)	Baseline *v*. Endline (MD = 1·06; 95 % CI (0·40, 1·70), *P*-value = 0·002)	Baseline *v*. Endline (MD = 0·01: 95 % CI (−0·31, 0·33), *P*-value = 0·930)
Habtmiriam *et al.*, 2022^(38)^	Percent change in proportion	Decreased from 43·1 % to 12·8 % in the intervention group but increased from 41·1 % to 49·5 % in the control group	Decreased from 12·1 % to 9·0 % in the intervention group but increased from 9·5 % to 36·0 % in the control group	Decreased from 15·4 % to 6·5 % in the intervention group but increased from 17·0 % to 68·6 % in the control group
Bidira *et al.*, 2022^(49)^	Difference in difference	Decreased from 45·6 % to 39·3 % in the intervention group but increased from 39·5 % to 41·2 % in the control group; DID = −0·08 (*P*-value > 0·050)	Decreased from 12·4 % to 6·9 % in the intervention group but increased from 13·9 % to 15·5 % in the control group; DID = −0·07 (*P*-value < 0·010)	Decreased from 23·7 % to 18·0 % in the intervention group but increased from 31·2 % to 33·2 % in the control group; DID = −0·08 (*P*-value < 0·001)
Ayalew *et al*., 2021^(39)^	Relative risk	RR = 0·68; 95 % CI (0·47, 0·99), *P*-value = 0·043	RR = 0·91; 95 % CI (0·49, 1·67), *P*-value = 0·752	RR = 0·55; 95 % CI (0·35, 0·87), *P*-value = 0·011
Han *et al*., 2021 ^(47)^	Percent change in proportion	Increased in the BCC group by 8·4 % in the vouchers group by 5·8 % and in the control group by 14·4 %, but a significant decrease was observed in BCC and vouchers group by 9·7 % (*P*-value < 0·05)	Decreased by 1·2 % in the BCC group and by 0·7 % in the control group but increased by 2·4 % in the vouchers group and by 3·2 % in the BCC and vouchers group	–
Abdulahi *et al*., 2021^(40)^	Difference in proportion	Stunting at endline was 4·8 % in the intervention group compared to 6·4 % in the control group (*P*-value = 0·565)	Wasting at endline was 4·0 % in the intervention group compared to 1·4 % in the control group (*P*-value = 0·051)	Underweight at endline was 3·2 % in the intervention group compared to 4·6 % in the control group (*P*-value = 0·618)
Teshome *et al*., 2020^(41)^	Percent change in proportion	Stunting increased in both intervention and control groups	Decreased from 14·4 % to 11·8 % in the intervention group but increased from 25·8 % to 29·7 % in the control group (*P*-value = 0·001)	Decreased from 32·9 % to 11·7 in the intervention group and from 34·0 % to 29·3 % in the control group (*P*-value = 0·001)
Passarelli *et al*., 2020^(42)^	Mean difference in Z-scores	ACGG *v*. Control (MD = 0·02; 95 % CI (−0·25, 0·29), *P*-value > 0·05); ACGG/ATONU *v*. Control (MD = 0·22; 95 % CI (−0·00, 0·45), *P*-value > 0·05); ACGG *v*. ACGG + ATONU (MD = 0·20; 95 % CI (−0·04, 0·45), *P*-value > 0·05)	ACGG *v*. Control (MD = −0·11; 95 % CI (−0·36, 0·14), *P*-value > 0·05); ACGG/ATONU *v*. Control (MD = −0·21; 95 % CI (−0·44, 0·05), *P*-value > 0·05); ACGG *v*. ACGG + ATONU (MD = −0·09; 95 % CI (−0·29, 0·10), *P*-value > 0·05)	ACGG *v*. Control (MD = 0·04; 95 % CI (−0·17, 0·25), *P*-value > 0·05); ACGG/ATONU *v*. Control (MD = −0·04; 95 % CI (−0·26, 0·17), *P*-value > 0·05; ACGG *v*. ACGG + ATONU (MD = −.0·08; 95 % CI (−0·27, 0·10), *P*-value > 0·05)
Mohammed *et al*., 2020^(43)^	B coefficients for Z-scores	Intervention *v*. Control (*β* = 0·06; 95 % CI (−0·13, 0·13), *P*-value > 0·05)	Intervention *v*. Control (*β* = −0·10: 95 % CI (−0·22, 0·05), *P*-value > 0·05)	Intervention *v*. Control (*β* = 0·01; 95 % CI (−0·13, 0·13), *P*-value > 0·05)
Kang *et al*., 2020^(75)^	Mean difference in Z-scores	Intervention *v*. Control (MD = 0·32; 95 % CI (0·08, 0·56), *P*-value < 0·001)	Intervention *v*. Control (MD = 0·48; 95 % CI (0·16, 0·81), *P*-value < 0·001)	Intervention *v*. Control (MD = 0·34; 95 % CI (0·06, 0·61), *P*-value <0·001)
Kim *et al*., 2019^(44)^	Mean difference in Z-scores	Intervention *v*. Control (MD = 0·12; 95 % CI (−0·09, 0·33), *P*-value > 0·05)	Intervention *v*. Control (MD = −0·09; 95 % CI (−0·33, 0·14), *P*-value > 0·05)	Intervention *v*. Control (MD = −0·02; 95 % CI (−0·20, 0·16), *P*-value > 0·05)
Samuel *et al*., 2018^(50)^	Difference in difference	Intervention *v*. Control (*β* = 0·18, se: 0·05, *P*-value < 0·005)	Intervention *v*. Control (*β* = −0·09, se: 0·05, *P*-value = 0·052)	Intervention *v*. Control (*β* = 0·01, se: 0·04, *P*-value = 0·780)
Argaw *et al*., 2018^(54)^	Mean difference in *Z*-scores	No significant difference between the control and intervention groups (*P*-value = 0·466)	MI *v*. Placebo (*β* = 0·005; 95 % CI (−0·012, 0·022), *P*-value = 0·538); CI *v*. Placebo (*β* = 0·022; 95 % CI (0·004, 0·039), *P*-value = 0·012); MCI *v*. Placebo (*β* = 0·018; 95 % CI (0·001, 0·034), *P*-value = 0·041)	–
Mulualem *et al*., 2016^(51)^	Mean difference in *Z*-scores	Intervention *v*. Control (*P*-value > 0·05)	Intervention *v*. Control (*P*-value < 0·05)	Intervention *v*. Control (*P*-value < 0·05)
Kim *et al*., 2016^(52)^	Difference in difference	Intervention *v*. Control (DID = 0·12, 95 % CI (0·09, 0·33), *P*-value > 0·05)	Intervention *v*. Control (DID = −0·09, 95 % CI (−0·33, 0·14), *P*-value > 0·05)	Intervention *v*. Control (DID = −0·02, 95 % CI (−0·20, 0·16), *P*-value > 0·05)
Akalu *et al*., 2010^(46)a^	Difference in difference	Intervention *v*. Control (*P*-value = 0·608)	Intervention *v*. Control (*P*-value = 0·048)	Intervention *v*. Control (*P*-value = 0·170)
Akalu *et al*., 2010^(46)b^	Difference in difference	Intervention *v*. Control (*P*-value = 0·015)	Intervention *v*. Control (*P*-value = 0·970)	Intervention *v*. Control (*P*-value = 0·013)
Fenn *et al*., 2011^(53)^	Difference in difference	Nutrition intervention *v*. Control (DID = 0·06; 95 % CI (−0·23, 0·18), *P*-value 0·620); Health intervention *v*. Control (DID = −0·16; 95 % CI (−0·43, 0·12), *P*-value = 0·230); WSH intervention *v*. Control (DID = 0·33; 95 % CI (0·08, 0·59), *P*-value = 0·020; Integrated intervention *v*. Control (DID = −0·03, 95 % CI (−0·47, 0·41), *P*-value = 0·880)	–	–

HAZ, height-for-age z-score; WHZ, weight-for-height *z*-score; WAZ, weight-for-age *z*-score; MD, mean difference; DID, difference in difference; RR, relative risk; BCC, behaviour change communication: ACGG, African Chicken Genetic Grains; ATONU, agriculture to nutrition.

A 12-month intervention using micronutrient powder supplementation for mothers/caregivers with children aged 6–12 months showed an improvement in children’s length-for-age *z*-score (*β* = 0·18, se: 0·05, *P*-value < 0·005)^(50)^. Additionally, an RCT demonstrated a positive impact on children’s height-for-age z-score when quality protein maize was provided to mothers/caregivers with children aged 7–56 months for 11 months (*P*-value = 0·015)^(46)^(Table 2). However, two studies showed an increase in the level of stunting after the implementation of BCC interventions^(39,41)^, and one study reported a decrease in the proportion of stunting but did not report statistical significance^(38)^. The remaining studies did not demonstrate positive impacts of relevant nutritional interventions on stunting^(42,44,49,51,52,54)^ (Table 2).

### Impacts of interventions on wasting/weight-for-height z-score

Six out of seventeen eligible studies reported the significant impacts of interventions on wasting/weight-for-height z-score. Among these six studies, two interventions targeting pregnant women did not demonstrate significant impacts on wasting. Mekonnen *et al.* conducted a 12-month nutritional education programme for mothers/caregivers with children aged less than a year that resulted in significant improvements in the weight-for length z-score of the children (MD = 1·06; 95 % CI (0·40, 1·70), *P*-value = 0·002)^(48)^. Bidira *et al.* showed improvements in wasting using nutritional education for mothers/caregivers with infants aged < 6 months of age (DID = -0·07; *P*-value < 0·010)^(49)^, and Teshome *et al.* demonstrated the effects of BCC on wasting for mothers/caregivers with children aged < 35 months of age (*P*-value = 0·001)^(41)^. Complementary feeding programmes (such as group nutrition sessions, cooking demonstrations, and supervision and training of healthcare workers) targeting mothers/caregivers with infants aged 6–12 months showed significant effects on the weight-for-height z-score of children (MD = 0·48; 95 % CI (0·16, 0·81), *P*-value < 0·001)^(45)^ (Table 2).

The implementation of a 6-month BCC intervention (including nutrition education, health belief model and recipe demonstrations) for mothers/caregivers with children aged 6–18 months resulted in a significant improvement in the weight-for-height z-score of children (*P*-value < 0·05)^(51)^. Providing fish oil supplementation for children (*β* = 0·022; 95 % CI (0·004, 0·039), *P*-value = 0·012), as well as for both mothers and children (*β* = 0·018; 95 % CI (0·001, 0·034), *P*-value = 0·041), improved the weight-for-length z-score of infants aged 6–12 months^(54)^. Except for two studies that did not report the level of statistical significance, the remaining studies did not show the significant impacts of interventions on wasting (Table 2).

### Impacts of interventions on underweight/weight-for-age *Z*-score

Out of fourteen studies, six reported significant impacts of interventions on underweight. Among these six studies, two studies implemented to target pregnant women did not show significant impacts. Bidira *et al.* implemented a nutritional education programme for mothers/caregivers with infants aged < 6 months of age, which resulted in a reduction in underweight (DID = -0·08, *P*-value < 0·001)^(49)^, while Ayalew *et al.* observed impacts on underweight through BCC interventions for the same age group (relative risk = 0·55; 95 % CI (0·35, 0·87), *P*-value = 0·011)^(39)^. Two studies that implemented BCC interventions indicated the significant impacts of interventions on underweight^(41,51)^. Kang *et al.* used a complementary feeding programme for mothers/caregivers with infants aged 6–12 months that reduced underweight (MD = 0·34; 95 % CI (0·06, 0·61), *P*-value <0·001)^(45)^. Providing quality protein maize for households with children under 5 years of age also improved the weight-for-age *z*-score of children (*P*-value = 0·013)^(46)^. However, except for two studies that did not report the level of statistical significance, the remaining studies did not show the impacts of interventions on underweight (Table 2).

## Discussion

Our study revealed that some BCC interventions, food vouchers, micronutrient supplementations and quality protein maize were effective in improving stunting. Similarly, BCC and fish oil supplementation also showed potential in reducing wasting, while BCC and the provision of quality protein maize reduced underweight. Furthermore, WaSH interventions that targeted pregnant mothers and children under 2 years of age were found to significantly reduce childhood stunting. Our searches did not locate any interventional study on the nutritional status of under 5 years of age children for Afar, Somali, Harari, Gambella and Benishangu regions, or Addis Ababa and Dire Dawa city administrations.

Recent global evidence demonstrated the importance of a systems-based approach to improve children’s diets and accelerate the reduction of malnutrition^(55,56)^. Our findings showed that almost two-thirds of published interventions in Ethiopia revealed no impact on childhood stunting and wasting, and more than half had no impact on underweight. Two explanations for this lack of impact are the short duration of intervention implementation, which may have limited their effectiveness, and the failure of most studies to recognise the importance of a systems-based approach. Crucially, integrating food (fruits, vegetables and animal foods), health (maternal and childcare), environment (clean water and adequate sanitation) and social protection (food vouchers) can all improve the nutritional status of children^(55)^. An appropriate systems-based approach requires a shared vision, joint planning and monitoring across stakeholders. Future child nutrition interventions in Ethiopia should adopt an integrated approach that combines the positive effects of interdependent systems such as BCC interventions, food programmes that boost protein and micronutrients, quality maternal and childcare interventions, clean water and adequate sanitation, and social protection systems (e.g. monetary support and income schemes).

Nutrition social and BCC strategies (such as interpersonal communication, social change approaches, community mobilisation, mass media and policy advocacy) encourage healthy behaviours while also reducing barriers to change^(57)^. Accordingly, interventional studies in Ethiopia have shown that BCC is a great measure to reduce childhood malnutrition^(32)^. Nevertheless, nutritional outcomes are also influenced by multiple factors, including household food security, individual behaviours, healthcare providers, school teachers, farmers, agricultural agents, religious and community leaders, private enterprises, and policymakers^(57,58)^. We, therefore, recommend that future BCC interventions in Ethiopia that aim to improve childhood malnutrition should consider locally specific factors that cover the households, communities and schools. Particular emphasis should also be given to implementing household food security interventions as a pivotal component of these strategies.

Animal-source foods (including meats, fish, poultry, organ meats and eggs) are rich sources of high-quality proteins and essential micronutrients^(59–61)^. Inadequate consumption of animal-source foods is substantially contributing to malnutrition and the suboptimal development of children in LMIC (including Ethiopia)^(62)^. In these countries, animal-source foods are not affordable to many low- and middle-income households and a significant number of families depend on starchy monotonous diets (e.g. grains, tubers and roots)^(63–65)^. Findings from eligible studies have shown the impacts of providing micronutrients and quality protein maize to households on stunting and wasting^(46,50)^. While micronutrient supplementation and plant proteins are important, they are not sustainable solutions for animal-source foods^(66)^. Ensuring access and affordability to animal-source foods (e.g. eggs, chicken and other livestock production) using national and subnational policy interventions can sustainably improve the consumption of animal-source foods for Ethiopian children.

Repeated childhood infections due to a lack of access to clean water and adequate sanitation contribute to the burden of malnutrition in LMIC, including Ethiopia^(67)^. In our review, Fenn *et al.* showed the impacts of WaSH interventions in the form of protected water supply and sanitation education on childhood stunting in Ethiopia^(53)^. However, recently published RCT documented a lack of impact of WaSH interventions on the nutritional status of children^(68–70)^. This lack of impact of WaSH interventions questioned the validity of the old hypothesis that WaSH has an independent effect on the linear growth of children^(71)^. Integrating WaSH interventions with food, health and social protection systems can successfully improve nutritional outcomes for children.

Reducing inequalities across regions and implementing effective strategies for malnutrition is essential to ensure that no child is left behind in Ethiopia. To date, no intervention studies on stunting, wasting and underweight have been published for Afar, Somali, Gambella and Benishangu regions, or Addis Ababa and Dire Dawa city administrations. Indeed, the government of Ethiopia has endorsed several global and national strategies to end malnutrition for the entire country; unfortunately, Addis Ababa, Dire Dawa and other city administrations have even higher rates of urbanisation driving the poor nutritional status of children in urban slums^(72,73)^. The observed inequalities warrant the need to expand successful interventions and strategies to reduce subnational inequalities for malnutrition.

The systematic review had some limitations. First, there was a wide heterogeneity of individual studies in intervention design and outcome measures, which ruled out the possibility of a meta-analysis. Second, nearly half of eligible studies were rated as high risk of bias (RCT and cluster RCT) or a serious risk of bias (quasi-experimental studies). The main reasons for not achieving a positive rating were the lack of allocation concealment methods, a higher number of concern decisions due to lack of information and the lack of RCT. To enhance the evidence base and ensure the reliability of future systematic reviews in this field, it is essential to conduct RCT with larger sample sizes and longer intervention durations.

Last, there is a chance of publication bias as studies showing negative results are less likely to be submitted for journal publication. Nevertheless, we conducted hand literature searches to locate grey literatures. Despite the above limitations, the study is the first systematic review of experimental and quasi-experimental studies to improve the nutritional status of children in Ethiopia. Our findings provide the best evidence for previously implemented interventions in Ethiopia.

### Conclusion

Our findings showed that almost two-thirds of published interventions had no impact on childhood stunting and wasting, and more than half had no impact on underweight. Future childhood nutritional interventions in Ethiopia should adopt an integrated approach that combines the positive effects of interdependent systems such as BCC interventions, food supplemental programmes (e.g. boosting protein and micronutrients), health interventions (e.g. strengthening maternal and childcare), clean water and adequate sanitation and social protection systems (e.g. monetary support and income schemes).

## Supporting information

Ahmed et al. supplementary material 1 Ahmed et al. supplementary material

Ahmed et al. supplementary material 2 Ahmed et al. supplementary material

Ahmed et al. supplementary material 3 Ahmed et al. supplementary material

## Supplementary material

For supplementary material accompanying this paper visit https://doi.org/10.1017/S1368980023002410
