# Supplementary material for: Interventions to improve the nutritional status of children under 5 years in Ethiopia: a systematic review
Source: Public Health Nutr. 2023 Oct 31;26(12):3147–61. doi: 10.1017/S1368980023002410 (PMC10755407; doi:10.1017/S1368980023002410)
Supplement: Ahmed et al. supplementary material 2 — Ahmed et al. supplementary material [file S1368980023002410sup002.pdf]

Edit Search

Search Name: Malnutrition_Ethiopia

Comment:

Save Cancel

Set	Search Statement	Annotations	Insert	Edit	Delete
1.	infant/ or infant, newborn/				
2.	child/ or child, preschool/				
3.	Female/				
4.	parents/ or fathers/ or mothers/				
5.	Caregivers/				
6.	(infan* or newborn* or new-born* or bab* or neonat* or perinat* or postnat* or kid* or toddler* or child* or young child* or p?ediatric* or mother* or female* or wom#n or caregiver* or care giver* or carer* or maternal).ab,ti.				
7.	1 or 2 or 3 or 4 or 5 or 6				
8.	Health Knowledge, Attitudes, Practice/				
9.	Health Education/				
10.	Health Promotion/				
11.	Counseling/				
12.	Health Literacy/				
13.	((breastfeed* adj2 (promot* or support* or education)) or (breast feed* adj2 (education or promot* or support)) or (health adj2 (education or literacy or promotion)) or ((nutrition* or food* or parent*or mother* or maternal) adj2 education) or ((nutrition* or parent or telephone) adj2 counsel*)).ti,ab.				
14.	Health Behavior/				
15.	Feeding Behavior/				
16.	health belief model/ or transtheoretical model/				
17.	communication/ or information seeking behavior/ or literacy/				
18.	Social Change/				
19.	Social Behavior/				
20.	social networking/ or online social networking/				
21.	peer group/ or peer influence/				
22.	Patient Advocacy/				
23.	"marketing of health services"/ or social marketing/				
24.	communications media/ or mass media/ or motion pictures/ or radio/ or television/ or pamphlets/ or social media/ or teaching materials/ or audiovisual aids/ or manuals as topic/ or textbooks as topic/ or electronic mail/ or telemedicine/ or telephone/ or videoconferencing/ or wireless technology/				
25.	((health adj2 (behavio?r or promot* behavio?r or related behavio?r)) or (behavio?r* adj2 (change or centered or change communication)) or communication or interpersonal communication or (information education and communication) or IEC or BCC or (social adj2 (change* or movement or mobiliz* or behavio?r or network* or market* or media)) or peer group* or advocacy or advocacy group* or (mass adj2 (communication or media)) or print media or (mobile adj2 (phone or health)) or mHealth or eHealth or electronic health or internet or radio or television or text messag* or SMS).ti,ab.				
26.	community health services/ or child health services/ or community health nursing/ or community mental health services/ or community networks/ or community participation/ or counseling/ or family planning services/ or foster home care/ or home care services/ or maternal health services/				
27.	social welfare/ or child welfare/ or community integration/ or infant welfare/ or maternal welfare/ or relief work/				
28.	Food Assistance/				
29.	preventive health services/ or early intervention, educational/ or immunization programs/ or primary prevention/ or secondary prevention/ or tertiary prevention/ or vaccination coverage/				
30.	(home visit or (community adj2 (program* or project* or intervention*or engagement* or leader or mobilization or gathering* or care service* or health action* or health service* or health care or role-play or health worker* or health aid* or health practitioner*)) or baby friendly community initiative* or baby-friendly community initiative* or demonstration* or cooking demonstration* or model breastfeeding communit* or auxiliary health worker* or health extension worker*).ti,ab.				
31.	health services/ or child care/ or infant care/ or facilitated tucking/ or kangaroo-mother care method/ or perinatal care/ or rooming-in care/ or community health services/ or child health services/ or early intervention, educational/ or maternal-child health services/ or home health nursing/ or community networks/ or community participation/ or community support/ or patient participation/				
32.	maternal health services/ or maternal-child health services/ or perinatal care/ or postnatal care/ or preconception care/ or prenatal care/				
33.	(((matern* or antenatal or prenatal or peri-natal or delivery or birth or home or neonatal or newborn or postnatal or postpartum or Kangaroo) adj2 care) or ((matern* or child or p?ediatric) adj2 health service*) or rooming-in-care or newborn nursery or essential nutrition action* or (home adj2 (visit* or help)) or homecare or caregiver* contact or baby-friendly or baby-friendly hospital initiative or BFHI or ANC or PNC or skin-to-skin contact or skin to skin contact or kangarooing or kangaroo mother care).ti,ab.				
34.	health services research/ or comparative effectiveness research/				
35.	evaluation study/				

36. Program Evaluation/
37. randomized controlled trial/
38. Non-Randomized Controlled Trials as Topic/
39. clinical trial/ or controlled clinical trial/
40. (effective* or impact* or evaluation or program* evaluation or project* or health project* or ((health or health care or healthcare) adj2 program*) or ((evaluation or experimental or interventional or quasi-experimental or quasi experimental or time-series or time series or random* controlled or cluster random*) adj2 stud*) or random* controlled design or random* controlled trial or RCT or random* clinical trial or cluster random* trial or cluster random* design or control* or placebo or comparison or ((usual or routine or standard) adj2 care)).ti,ab.
41. 8 or 9 or 10 or 11 or 12 or 13 or 14 or 15 or 16 or 17 or 18 or 19 or 20 or 21 or 22 or 23 or 24 or 25 or 26 or 27 or 28 or 29 or 30 or 31 or 32 or 33 or 34 or 35 or 36 or 37 or 38 or 39 or 40
42. malnutrition/ or deficiency diseases/ or fetal nutrition disorders/ or severe acute malnutrition/ or kwashiorkor/ or starvation/
43. Growth Disorders/
44. Wasting Disease, Chronic/
45. Thinness/
46. nutrition disorders/ or child nutrition disorders/ or infant nutrition disorders/
47. (stunt* or wast* or underweight or undernutrition or malnutrition or thin*).ti,ab.
48. nutritional physiological phenomena/ or child nutritional physiological phenomena/ or infant nutritional physiological phenomena/ or diet/ or hunger/
49. Child Development/
50. (stunt* or wast* or underweight or undernutrition or malnutrition or thin* or hunger or child* growth or child* development or nutritional status).ti,ab.
51. 42 or 43 or 44 or 45 or 46 or 47 or 48 or 49 or 50
52. Ethiopia.mp. or Ethiopia/
53. 7 and 41 and 51 and 52

Save Cancel

English Français Italiano Deutsch 日本語 繁體中文 Español 简体中文 한국어

About Us Contact Us Privacy Policy Terms of Use

© 2022 Ovid Technologies, Inc. All rights reserved. OvidUI_04.20.01.001, SourceID bab09fc86e1540d01875b74c93f5fdf6f33181f0
