# Supplementary material for: Interventions to improve the nutritional status of children under 5 years in Ethiopia: a systematic review
Source: Public Health Nutr. 2023 Oct 31;26(12):3147–61. doi: 10.1017/S1368980023002410 (PMC10755407; doi:10.1017/S1368980023002410)
Supplement: Ahmed et al. supplementary material 3 — Ahmed et al. supplementary material [file S1368980023002410sup003.docx]

**Excluded studies**

The main reasons for the exclusion of 60 studies included: 29 studies did not measure the required anthropometric outcomes ^(1-29)^, 13 were not interventional studies ^(30-42)^, 6 studies did not design for under-5 children ^(43-48)^, 10 studies were conference abstracts ^(49-58)^, 1 study described the implementation process ^(59)^, and 1 study was duplicate ^(60)^.

1. Workicho A, Biadgilign S, Kershaw M *et al.* (2021) Social and behaviour change communication to improve child feeding practices in Ethiopia. *Maternal & child nutrition* 17, e13231.

2. Solomon ET, Gari SR, Kloos H *et al.* (2021) Handwashing effect on diarrheal incidence in children under 5 years old in rural eastern Ethiopia: a cluster randomized controlled trial. *Tropical Medicine & Health* 49, 1-11.

3. Sisay G & Tesfaye A (2021) Effects of nutrition education and counseling intervention on maternal weight and obstetric outcomes among pregnant women of Gedeo Zone, Southern, Ethiopia: A cluster randomized control trial. *Nutrition and health*, 2601060211056745.

4. Mohammed H, Marquis GS, Aboud F *et al.* (2021) TSH Mediated the Effect of Iodized Salt on Child Cognition in a Randomized Clinical Trial. *Nutrition & Metabolic Insights*, 1-9.

5. LaFave D, Beyene AD, Bluffstone R *et al.* (2021) Impacts of improved biomass cookstoves on child and adult health: Experimental evidence from rural Ethiopia. *World Development* 140, N.PAG-N.PAG.

6. Avan BI, Berhanu D, Mekonnen Y *et al.* (2021) Embedding Community-Based Newborn Care in the Ethiopian health system: lessons from a 4-year programme evaluation. *Health policy and planning* 36, i22-i32.

7. Ambikapathi R, Passarelli S, Madzorera I *et al.* (2021) Men's nutrition knowledge is important for women's and children's nutrition in Ethiopia. *Maternal & child nutrition* 17, e13062.

8. Demilew YM, Alene GD Belachew T (2020) Effect of guided counseling on dietary practices of pregnant women in West Gojjam Zone, Ethiopia. *PloS one* 15, e0233429.

9. Demilew YM, Alene GD Belachew T (2020) Effects of guided counseling during pregnancy on birth weight of newborns in West Gojjam Zone, Ethiopia: a cluster-randomized controlled trial. *BMC pediatrics* 20, 466.

10. Abiyu C & Belachew T (2020) Effect of complementary feeding behavior change communication delivered through community-level actors on dietary adequacy of infants in rural communities of West Gojjam Zone, Northwest Ethiopia: A cluster-randomized controlled trial. *PloS one* 15, e0238355.

11. Abiyu C & Belachew T (2020) Effect of complementary feeding behavior change communication delivered through community-level actors on the time of initiation of complementary foods in rural communities of West Gojjam zone, Northwest Ethiopia: a cluster-randomized controlled trial. *BMC pediatrics* 20, 509.

12. Keenan JD, Gebresillasie S, Stoller NE *et al.* (2019) Linear growth in preschool children treated with mass azithromycin distributions for trachoma: A cluster-randomized trial. *PLoS neglected tropical diseases* 13, e0007442.

13. Argaw A, Huybregts L, Wondafrash M *et al.* (2019) Neither n-3 Long-Chain PUFA Supplementation of Mothers through Lactation nor of Offspring in a Complementary Food Affects Child Overall or Social-Emotional Development: A 2 x 2 Factorial Randomized Controlled Trial in Rural Ethiopia. *The Journal of nutrition* 149, 505-512.

14. Alemu F, Mecha M Medhin G (2019) Impact of permagarden intervention on improving fruit and vegetable intake among vulnerable groups in an urban setting of Ethiopia: A quasi-experimental study. *PloS one* 14, e0213705.

15. Worku BN, Abessa TG, Wondafrash M *et al.* (2018) Effects of home-based play-assisted stimulation on developmental performances of children living in extreme poverty: a randomized single-blind controlled trial. *BMC pediatrics* 18, 29.

16. Kung'u JK, Pendame R, Ndiaye MB *et al.* (2018) Integrating nutrition into health systems at community level: Impact evaluation of the community-based maternal and neonatal health and nutrition projects in Ethiopia, Kenya, and Senegal. *Maternal & child nutrition* 14 Suppl 1.

17. Karim AM, Fesseha Zemichael N, Shigute T *et al.* (2018) Effects of a community-based data for decision-making intervention on maternal and newborn health care practices in Ethiopia: a dose-response study. *BMC Pregnancy & Childbirth* 18, N.PAG-N.PAG.

18. Bougma K, Aboud FE, Lemma TM *et al.* (2018) Introduction of iodised salt benefits infants' mental development in a community-based cluster-randomised effectiveness trial in Ethiopia. *The British journal of nutrition* 119, 801-809.

19. Yunhee K, Youn Kyoung S, Debele L *et al.* (2017) Effects of a community-based nutrition promotion programme on child feeding and hygiene practices among caregivers in rural Eastern Ethiopia. *Public Health Nutrition* 20, 1461-1472.

20. Buchanan SE & Pose B (2012) Improving child survival through behavioral change and community engagement: the Farta, Ethiopia Child Survival Project. *International Journal of Health Promotion & Education* 50, 145-158.

21. Coppock DL, Desta S, Tezera S *et al.* (2011) Capacity building helps pastoral women transform impoverished communities in Ethiopia. *Science (New York, NY)* 334, 1394-1398.

22. Klein PS & Rye H (2004) Interaction-oriented early intervention in Ethiopia: the MISC approach...More Intelligent and Sensitive Children. *Infants & Young Children: An Interdisciplinary Journal of Early Childhood Intervention* 17, 340-354.

23. Muluye SD, Lemma TB Diddana TZ (2020) Effects of Nutrition Education on Improving Knowledge and Practice of Complementary Feeding of Mothers with 6- to 23-Month-Old Children in Daycare Centers in Hawassa Town, Southern Ethiopia: An Institution-Based Randomized Control Trial. *Journal of Nutrition & Metabolism*, 1-10.

24. Tariku B, Whiting SJ, Mulualem D *et al.* (2015) Application of the Health Belief Model to Teach Complementary Feeding Messages in Ethiopia. *Ecology of food and nutrition* 54, 572-582.

25. Negash C, Belachew T, Henry CJ *et al.* (2014) Nutrition education and introduction of broad bean-based complementary food improves knowledge and dietary practices of caregivers and nutritional status of their young children in Hula, Ethiopia. *Food and nutrition bulletin* 35, 480-486.

26. Carnell MA, Dougherty L, Pomeroy AM *et al.* (2014) Effectiveness of scaling up the 'three pillars' approach to accelerating MDG 4 progress in Ethiopia. *Journal of health, population, and nutrition* 32, 549-563.

27. Mengistie B, Berhane Y Worku A (2013) Household Water Chlorination Reduces Incidence of Diarrhea among Under-Five Children in Rural Ethiopia: A Cluster Randomized Controlled Trial. *PloS one* 8, 1-7.

28. Guyon A, Mehdin M Jirga A (2013) Improving complementary feeding practices through the essential nutrition actions (ENA) framework in Southern Ethiopia. *Annals of Nutrition and Metabolism* 63, 604.

29. Cherkos TM, Hailemichael T, Sinamo S *et al.* (2013) Role of nutrition education to overcome food taboos and improve iron tablets demand during pregnancy in ethiopia: Wonchi district. *Annals of Nutrition and Metabolism* 63, 1738.

30. Swanson V, Hart J, Byrne-Davis L *et al.* (2021) Enhancing Behavior Change Skills in Health Extension Workers in Ethiopia: Evaluation of an Intervention to Improve Maternal and Infant Nutrition. *Nutrients* 13.

31. Rokicki S (2021) Impact of family law reform on adolescent reproductive health in Ethiopia: A quasi-experimental study. *World Development* 144, N.PAG-N.PAG.

32. Oladeji O, Oladeji B, Diaaeldin Omer M *et al.* (2021) Exploring opportunities to enhance effectiveness of mobile health and nutrition strategy for providing health and nutrition services amongst pastoralists in Somali region, Ethiopia. *African journal of primary health care & family medicine* 13, e1-e7.

33. Bahru BA, Jebena MG, Birner R *et al.* (2020) Impact of Ethiopia's productive safety net program on household food security and child nutrition: A marginal structural modeling approach. *SSM - Population Health* 12, 100660.

34. J. R H, H P, W T *et al.* (2019) INTEGRATION OF WATER, SANITATION, HYGIENE AND NUTRITION PROGRAMMING IS ASSOCIATED WITH LOWER PREVALENCE OF CHILD STUNTING AND FEVER IN OROMIA, ETHIOPIA. *African Journal of Food, Agriculture, Nutrition & Development* 19, 14971-14977.

35. Gebrehiwot T & Castilla C (2019) Do Safety Net Transfers Improve Diets and Reduce Undernutrition? Evidence from Rural Ethiopia. *Journal of Development Studies* 55, 1947-1966.

36. Baye K, Retta N Abuye C (2014) Comparison of the effects of conditional food and cash transfers of the Ethiopian Productive Safety Net Program on household food security and dietary diversity in the face of rising food prices: ways forward for a more nutrition-sensitive program. *Food and nutrition bulletin* 35, 289-295.

37. Zello GA, Whiting SJ, Kebebu A *et al.* (2013) Increasing pulse intake to reduce malnutrition in traditional pulse growing regions in southern ethiopia. *Annals of Nutrition and Metabolism* 63, 1757-1758.

38. Tarigan M, Gedefaw M Wilder-Smith A (2013) Impact of model household training by health extension workers on immunization coverage, nutritional status, and insecticide treated-nets utilization in Dera District-Ethiopia. *Tropical Medicine and International Health* 18, 201.

39. Berti PR, Mildon A, Siekmans K *et al.* (2010) An adequacy evaluation of a 10-year, four-country nutrition and health programme. *International journal of epidemiology* 39, 613-629.

40. Richard DS, Saskia de P, Kai S *et al.* (2008) Coverage of the National Vitamin A Supplementation Program in Ethiopia. *Journal of Tropical Pediatrics* 54, 141-141.

41. Quisumbing AR (2003) Food Aid and Child Nutrition in Rural Ethiopia. *World Development* 31, 1309.

42. Haidar J, Tsegaye D, Mariam DH *et al.* (2003) Vitamin A supplementation on child morbidity. *East African medical journal* 80, 17-21.

43. Nane D, Hatloy A Lindtjorn B (2021) A local-ingredients-based supplement is an alternative to corn-soy blends plus for treating moderate acute malnutrition among children aged 6 to 59 months: A randomized controlled non-inferiority trial in Wolaita, Southern Ethiopia. *PloS one* 16, e0258715.

44. Dansa R, Reta F, Mulualem D *et al.* (2019) A Nutrition Education Intervention to Increase Consumption of Pulses Showed Improved Nutritional Status of Adolescent Girls in Halaba Special District, Southern Ethiopia. *Ecology of food and nutrition* 58, 353-365.

45. Abessa TG, Worku BN, Wondafrash M *et al.* (2019) Effect of play-based family-centered psychomotor/psychosocial stimulation on the development of severely acutely malnourished children under six in a low-income setting: a randomized controlled trial. *BMC pediatrics* 19, 336.

46. Whiting S, Berhanu G, Henry C *et al.* (2017) Effect of nutrition education to mothers on improved pulse consumption by young children aged 6-24 months in rural Sidama, South Ethiopia. *Annals of Nutrition and Metabolism* 71, 1271.

47. Aboud FE, Bougma K, Lemma T *et al.* (2017) Evaluation of the effects of iodized salt on the mental development of preschool-aged children: a cluster randomized trial in northern Ethiopia. *Maternal & child nutrition* 13.

48. Walker CLF, Bhutta ZA, Bhandari N *et al.* (2007) Zinc during and in convalescence from diarrhea has no demonstrable effect on subsequent morbidity and anthropometric status among infants < 6 mo of age. *American Journal of Clinical Nutrition* 85, 887-894.

49. Whiting S, Omer A, Mulualem D *et al.* (2017) Feasibility study of an intervention of gifting chickens for young children in ethiopia for provision of egg and eggshell. *Annals of Nutrition and Metabolism* 71, 402.

50. Samuel A, Osendarp S, Adish AA *et al.* (2017) Effectiveness of a program intervention with reduced-iron MNPS on morbidity, iron status and child growth in young children in Ethiopia. *Annals of Nutrition and Metabolism* 71, 837-838.

51. Mohammed H, Marquis G, Aboud F *et al.* (2017) Effect of early market introduction of iodized salt on pregnancy and birth outcomes in a randomized clinical trial in the Amhara region of Ethiopia. *FASEB Journal* 31.

52. Henry C, Fereja M, Mesfin A *et al.* (2017) Empowering women & women-headed households through production, nutrition education and consumption strategies: lessons learned from pulsebased nutrition sensitive agricultural strategies in Ethiopia. *Annals of Nutrition and Metabolism* 71, 214.

53. Ersino G, Henry CJ Zello GA (2017) A nutrition education intervention affects the diet-health related practices and nutritional status of mothers and children in a pulse-growing community in Halaba, south Ethiopia. *FASEB Journal* 31.

54. Kang Y, Sinamo S, Kim S *et al.* (2015) Effectiveness of a communitybased participatory nutrition promotion program to improve linear and ponderal growth in children 6 to 24 months of age in rural Eastern Ethiopia: A cluster randomized trial. *FASEB Journal* 29.

55. Bougma K, Marquis G, Aboud F *et al.* (2014) Iodized salt benefits infant mental development: Preliminary results from a cluster randomized trial in Ethiopia. *FASEB Journal* 28.

56. Reider K, Beyero M Mekonnen Y (2013) Effect of timed and targeted counseling (TTC) in changing infant and young child feeding practices in Southern Ethiopia. *Annals of Nutrition and Metabolism* 63, 629.

57. Beyero M, Reider K Mekonnen Y (2013) Effect of timed and targeted counseling (ttC) by peer mothers in changing infant and young child feeding practices among mothers of children 0 to 18 months in four districts in Southern Ethiopia. *FASEB Journal* 27.

58. Stoller NE, Amza A, Boubacar K *et al.* (2012) Evaluating the effects of mass azithromycin distributions on childhood growth and nutrition: Lessons learned from two pilot studies. *American Journal of Tropical Medicine and Hygiene* 87, 10.

59. Kang Y, Cha S, Yeo S *et al.* (2017) Implementation, utilization and influence of a community-based participatory nutrition promotion programme in rural Ethiopia: programme impact pathway analysis. *Public health nutrition* 20, 2004-2015.

60. Kang Y, Suh YK, Debele L *et al.* (2017) Effects of a community-based nutrition promotion programme on child feeding and hygiene practices among caregivers in rural Eastern Ethiopia. *Public health nutrition* 20, 1461-1472.
